# Problem Solving Therapy Improves Effortful Cognition in Major Depression

**DOI:** 10.3389/fpsyt.2021.607718

**Published:** 2021-04-07

**Authors:** Chenguang Jiang, Hongliang Zhou, Limin Chen, Zhenhe Zhou

**Affiliations:** ^1^Wuxi Mental Health Center Affiliated to Nanjing Medical University, Wuxi, China; ^2^Nanjing Brain Hospital Affiliated to Nanjing Medical University, Nanjing, China

**Keywords:** major depression, problem solving therapy, effortful cognition, the face-vignette task, cognitive function

## Abstract

**Background:** Effortful cognition processing is an intentionally initiated sequence of cognitive activities, which may supply top-down and goal-oriented reassessment of specific stimuli to regulate specific state-driven responses contextually, whereas automatic cognitive processing is a sequence of cognitive activities that is automatically initiated in response to an input configuration. The effortful–automatic perspective has implications for understanding the nature of the clinical features of major depressions. The aim of this study was to investigate the influence of problem solving therapy (PST) on effortful cognition in major depression (MD).

**Methods:** The participants included an antidepressant treatment (AT) group (*n* = 31) or the combined antidepressant treatment and PST (CATP) group (*n* = 32) and healthy controls (HCs) (*n* = 30). Hamilton Depression Rating Scale (HAMD, 17-item version) and the face–vignette task (FVT) were measured for AT group and CATP group at baseline (before the first intervention) and after 12 weeks of interventions. The HC group was assessed with the FVT only once. At baseline, both patients and HCs were required to complete the basic facial emotion identification test (BFEIT).

**Results:** The emotion identification accuracy of the HC group was higher than that of the patient group when they performed BFEIT; patients with MD present poor FVT performances; compared to the antidepressant treatment, PST plus antidepressant treatment decreased HAMD scores and improved FVT performances in patients with MD.

**Conclusions:** Patients with MD present effortful cognition dysfunction, and PST can improve effortful cognitive dysfunction. These findings suggest that the measurement of effortful cognition might be one of the indexes for the therapeutic effect of PST in MD.

## Introduction

Major depression (MD) is a common mental disorder with a higher disability rate, affecting 10–15% of the worldwide population every year. To date, some antidepressants, including several typical antidepressants and several atypical antidepressants, have been used to treat major depression; however, only 60–70% of patients respond to antidepressant treatment. Furthermore, 10–30% of these patients exhibit treatment-resistant symptoms such as suicidal thought, a low mood, a decline in interest, and a loss of happiness (1).

To improve the symptoms of MD, several treatment options have been developed, such as switching therapies, augmentation, combination, optimization, psychotherapies, modified electro-convulsive therapy (MECT), repetitive transcranial magnetic stimulation therapies, deep brain stimulation therapies, vagal nerve stimulation therapies, light-based therapies, acupuncture treatment, and yoga; these approaches have been considered and tailored for individual patients (2–4). Most important for the improvement of depressed patients' symptoms, many studies had reported that physical activity interventions are helpful to improve major depressive disorders because physical activity is associated with many mental health benefits (5–11). Assessments to determine symptom improvement for patients with MD often depend on decreased total Hamilton Depression Rating Scale (HAMD, 17 or 24 items) scores.

Problem solving therapy (PST) belongs to a type of cognitive behavioral therapy that mainly concentrates on training in appropriate problem-solving notions as well as skills. PST has been used for major depression (12–15). It has been confirmed that, in the depressed patient group, PST was equally effective as antidepressant treatments and more effective than no treatment and support or attention control patients (16). In clinical practice, the effective treatment program of PST in MD includes three aspects: [1] training in a positive problem orientation, [2] training in problem definition and formulation, the generation of alternatives, decision making, and solution implementation and verification, and [3] training in problem orientation plus problem definition and formulation, the generation of alternatives, decision making, and solution implementation and verification (16).

Cognitive function refers to mental processes involved in working memory, problem-solving, decision-making, the acquisition of knowledge, regulation of information, and reasoning. As a major symptom, cognitive function impairment is acknowledged as a clinical characteristic of major depression. Additionally, many studies of major depression have suggested a role for cognitive measures in predicting those at risk for poor outcomes (17). A previous study indicated that patients with major depression present negatively valanced emotional symptoms that are accompanied by cognitive deficits, and the emotional processing dysfunctions of the prefrontal cortex might lead to cognitive deficits in patients with MD (18). Adaptive emotional responding relies on both effortful cognition processing and automatic cognition processing. Effortful cognition processing is a controlled process and refers to an intentionally initiated sequence of cognitive activities, which may supply top-down as well as goal-oriented reassessment of emotional stimuli to regulate emotion-driven responses contextually (19). Effortful cognition was measured by the face–vignette task (FVT) (19). Relative to effortful cognitive processing, automatic cognitive processing is a sequence of cognitive activities that is automatically initiated in response to an input configuration (20). Automatic cognition processing requires near-zero attention for the task at hand and, in many instances, is executed in response to a specific stimulus.

Previous studies have shown that patients with MD present effortful cognitive dysfunction. For example, a previous study reported that, when patients with MD performed two contrasting cognitive tasks (*i.e*., one requiring sustained effort and information processing and the other requiring only superficial information processing that could be accomplished automatically), only the effort-demanding cognitive task was performed poorly (21). Additionally, two previous studies investigated the functions of automatic and effortful information processing in a visual search paradigm, and the results showed that the patients with MD exhibited longer reaction times on the tasks requiring more effortful information processing than the controls. However, there were no differences on tasks requiring automatic information processing (22, 23).

Since cognitive function impairment plays a critical role in MD, the assessment of cognitive function is a better way to determine the treatment effect for MD. The effortful–automatic perspective has implications for understanding the nature of the clinical features of MD. Furthermore, the investigation of the influence of PST on effortful cognition in MD is helpful for improving the present understanding of the therapeutic mechanism and assess the therapeutic effect of PST. To date, no studies of PST on effortful cognition in MD have been reported. In this study, the participants included patients with MD and healthy controls (HCs). The MD group was treated with antidepressants or the combination of antidepressants with PST, and effortful cognition was rated by the FVT. The hypothesis of this study is that depressed patients display poor effortful cognition performance, and PST can improve effortful cognitive dysfunctions. The aim of this study was to investigate the effect of PST on effortful cognition in MD.

## Materials and Methods

### Time and Setting

This study was conducted in Wuxi Mental Health Center Affiliated to Nanjing Medical University, No. 156 Qianrong Road, Rongxiang Street, Binhu District, Wuxi City, P.R. China, from February 1, 2016 to February 27, 2020.

### Diagnostic Approaches and Subjects

A total of 80 patients meeting the American Psychiatric Association's fifth edition of the Diagnostic and Statistical Manual of Mental Disorders (DSM-5) criteria for major depression were recruited as the research group. The MD patients were randomly assigned to the antidepressant treatment (AT) group or the combined antidepressant treatment and PST (CATP) group. The allocation schedule was generated by using a list of random numbers. Thirty healthy persons were admitted to the HC group. All HCs had no personal history of mental disorders. Patients with MD were selected from Wuxi Mental Health Center Affiliated to Nanjing Medical University, No. 156 Qianrong Road, Rongxiang Street, Binhu District, Wuxi City, P.R. China; the normal controls were citizens of Wuxi City, Jiangsu Province, P.R. China, recruited by online and local community advertisements. Patients with MD and HC subjects were excluded from the study if they had been diagnosed with nicotine addiction or other psychoactive substance dependence, had suffered any systemic disease that may affect the central nervous system, or had received electroconvulsive therapy (including MECT) in the past 24 weeks. All patients and HC subjects were Chinese. All patients and HC subjects were paid 42.12 Euros plus travel costs.

Seven subjects in AT group and five subjects in CATP group were all diagnosed with bipolar disorder in the follow-up survey, and they were ultimately excluded from this study. Two subjects in AT group and three subjects in CATP group were also excluded from this study because they could not finish the follow-up assessment. Finally, the data from 31 subjects in AT group and 32 subjects in CATP group were used in the statistical analyses.

### Measurements of Automatic and Effortful Cognition

#### Basic Facial Emotion Identification Test

The basic facial emotion identification test (BFEIT) consists of eight examples of each of the seven basic facial emotions, *e.g*., happy, angry, sad, fear, surprise, disgust, and calm, which were taken from the Chinese affective picture system (24). Male and female face pictures were balanced across each emotion category.

### Face–Vignette Task

**Figure 1:**
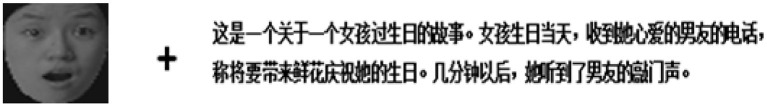
Example of a trial on the face–vignette task. The situational vignettes in English are as follows: This is a story about a girl's birthday. The girl stayed in her room. She received a call from her beloved boyfriend: “You're waiting for me at home. I'll bring your favorite flowers to your birthday!” Several minutes later, she heard the knock of her boyfriend's arrival. The question was “What emotion is the person feeling?” Responding with “surprise” will be recorded as a face response and responding with “hopeful” will be recorded as a vignette response. Additionally, any other response will be recorded as a random response.

FVT was designed based on an effortful cognitive task that was used in the study on effortful *vs*. automatic emotional processing in patients with schizophrenia by Patrick et al. (19). E-Prime 2.0 software (Psychology software tools, INC, USA) was used to implement the experimental procedure. The face pictures were white and black photographs and included six emotional expressions, *i.e*., happy, angry, sad, fear, surprise, and disgust, which were taken from the Chinese affective picture system (24). In each emotion, the male and female faces were equal. Within a given emotion category, the same identity was used only once. The situational vignettes communicated the six special emotions, *i.e*., guilty, smug, hopeful, insulted, pain, and determined. Before the experiment, the intended emotion for each story (vignette) was verified by seven undergraduates, and the mean accuracy was 0.91 [standard deviation (SD) = 0.08], and the observed inter-rater reliability κ value was 0.75. The face–story pairs were matched such that each story was inconsistent with the facial expression according to the specially appointed emotional category (*e.g*., a happy facial expression paired with a smug story). Each specific emotion category depended on the situational context (see the listed example in Figure 1). The specially appointed face–story pairs included sad *vs*. guilty, happy *vs*. smug, fearful *vs*. painful, angry *vs*. determined, disgusted *vs*. insulted, and surprised *vs*. hopeful. During the FVT, the participants viewed a series of 24 face–story (vignette) pairs and were informed that each facial expression represented the subject of the vignette. The faces and vignettes were presented simultaneously. All participants were required to read the vignettes aloud. In each trial, all participants answered the question accompanied by face–vignette pairs through a specially appointed keypad in a multiple choice pattern. The 13 obtainable choices for each trial were as follows: angry, happy, sad, fearful, disgusted, surprised, smug, guilty, hopeful, determined, pain, insulted as well as no emotion.

On the FVT, the responses of the participants were labeled as face responses, vignette responses, and random responses. The response data were converted to proportions, which were used for statistical analysis.

### Problem Solving Therapy Procedure

The PST was performed as described in a previous study (25). All the patients with MD were scheduled for PST, which consists of six sessions administered every other week. The treatment sessions were conducted at the psychological therapy room of the Psychiatry Department. The PST was conducted by six psychotherapists, and visits were conducted by two psychiatric resident physicians. All the psychotherapists owned a therapy handbook and underwent training, including a short theoretical course, role playing in a clinical background as well as watching a training videotape. The PST includes three steps: [1] the patient's symptoms are linked with their problems in daily living, [2] the problems are defined and clarified, and [3] an attempt is made to solve the problems in a structured way. The sessions lasted 1 h for the first visit and half an hour for the subsequent visits.

### Clinical Interventions and Clinical Assessment

Two psychiatric residents examined all the participants to confirm or exclude a major depression diagnosis based on DSM-5 criteria and to collect medication and sociodemographic data. A HAMD (17-item version) was applied to assess the depressive severity for patients. A decrease of more than 50% in HAMD (17-item version) scores from baseline to follow-up was defined as a treatment response, and HAMD (17-item version) scores <7 at follow-up were defined as clinical remission.

HAMD (17-item version) and the FVT data were measured for the AT group and CATP group at baseline (before the first intervention, time 1) and after 12 weeks of interventions (time 2). The HC group was assessed using the face–vignette task only once. At baseline, both patients and HCs were required to complete the BFEIT.

### Statistical Analysis

Data are presented as mean (SD), and all data were analyzed with Statistical Product and Service Solution 18.0 statistical software (SPSS 18.0, International Business Machines Corporation). Comparisons of the demographic data, basic facial emotion identification test scores, face response proportions, vignette response proportions, and random response proportions at baseline among patients and healthy controls were conducted using the method of one-way analysis of variance (ANOVA) or the chi-square test. Comparisons of HAMD (17-item version) scores, face response proportions, vignette response proportions, and random response proportions between baseline (time 1) and after 12 weeks of interventions (time 2) in the patient group were performed using 2 × 2 repeated-measures ANOVA. In this study, all alpha values of 0.05 were considered as statistically significant throughout. Cohen's *d* effect sizes were used for *t*-tests. The cutoff values for Cohen's *d*'s were defined as trivial effect size when *d* < 0.19, small effect size when 0.2 < *d* < 0.49, medium effect size when 0.5 < *d* < 0.79, and large effect size when *d* > 0.8. Partial eta-square (η_*p*_^2^) effect sizes were used for *F*-tests. Similarly, the cutoff values for η_*p*_^2^ were set as trivial effect size when η_*p*_^2^ < 0.019, small effect size when 0.02 < η_*p*_^2^ < 0.059, medium effect size when 0.06 < η_*p*_^2^ < 0.139, and large effect size when η_*p*_^2^ > 0.14. Phi (ϕ) effect sizes were used for chi-square test. The cutoff values for ϕ were set as trivial effect size when ϕ < 0.09, small effect size when 0.10 < ϕ < 0.29, medium effect size when 0.30 < ϕ < 0.49, and large effect size when ϕ > 0.50.

## Results

### The Demographic Data of All Participants

The demographic data of the participants are described in Table 1. No significant differences were observed in sex ratio, mean age, age range, or mean education years among the AT group, CATP group, and HC group.

**Table 1:** Demographic characteristics and clinical data of all participants.

	**AT group**	**CATP group**	**HC group**	**Test statistic**	***Post hoc* analyses**
Sex ratio (male/female)	31 (15/16)	32 (16/16)	30 (15/14)	χ^2^ = 6.567, *p* = 0.146, not significant	–
Mean age (SD)	35 (14)	36 (14)	35 (13)	*F*_2,90_ = 4.690, *p* = 0.679, *η_*p*_*^2^ **=** 0.000, not significant	–
Age range	18–53	19–53	18–54	–	–
Education (SD)	8.3 (3.2)	8.2 (3.3)	9.1 (3.0)	*F*_2,90_ = 3.786, *p* = 0.679, *η_*p*_*^2^ **=** 0.001, not significant	–
Duration (month, SD)	3.1 (3.0)	3.5 (3.0)	–	*t =* 2.356, Cohen's *d* = 0.001, *p =* 0.236	–

*AT, antidepressant treatment; CATP, the combination of antidepressant treatment and PST; HC, healthy control; SD, standard deviation; $\eta_{\text{p}}^{2}$, partial eta-square*.

### Antidepressant Treatments

In the AT group, 20 patients with MD were antidepressant-naïve, and 11 patients with MD were antidepressant-free (six for at least 24 weeks and five for at least 4 weeks); patients with MD received fluoxetine (*n* = 8), paroxetine (*n* = 7), fluvoxamine (*n* = 7), sertraline (*n* = 6), or escitalopram (*n* = 3). The mean fluoxetine-equivalent dose was 30.5 (8.8) mg/day. In the CATP group, 19 patients with MD were antidepressant-naïve, and 13 patients with MD were antidepressant-free (eight for at least 24 weeks and five for at least 4 weeks); patients with MD received fluoxetine (*n* = 9), paroxetine (*n* = 8), fluvoxamine (*n* = 8), sertraline (*n* = 3), or escitalopram (*n* = 4). According to a previous report (26), the mean fluoxetine-equivalent dose was 30.1 (7.9) mg/day. Neither of the patient groups used concomitant medications.

### Comparisons of BFEIT Performance Among the AT Group, CATP Group, and HC Group

**Figure 2:**
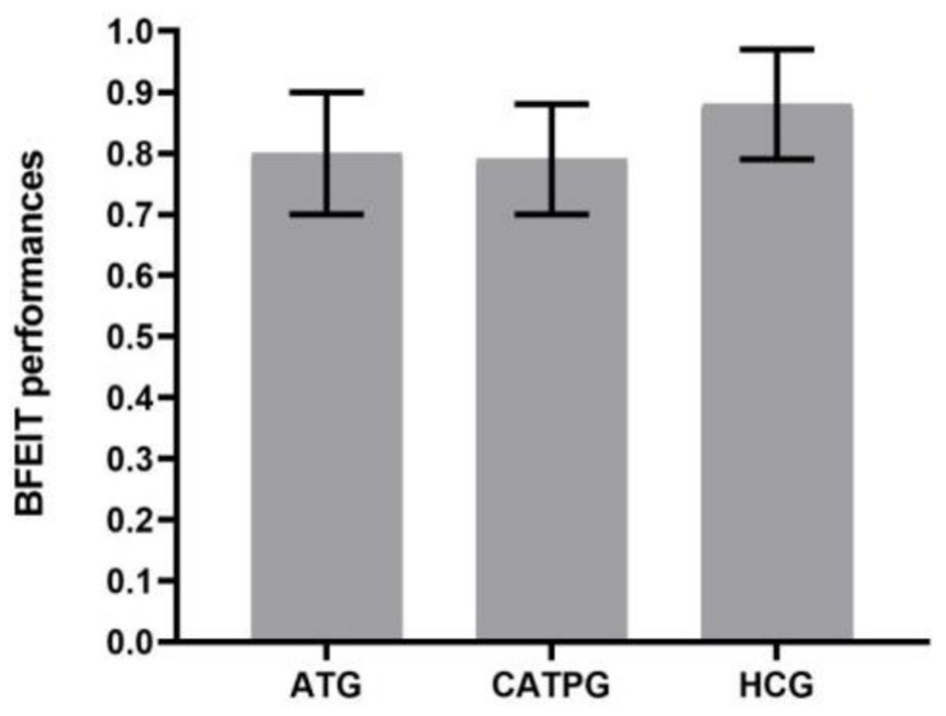
Comparisons of BFEIT performance among the AT group, CATP group, and HC group. BFEIT, basic facial emotion identification test; ATG, antidepressant treatment group; CATPG, the combination of antidepressant treatment and PST group; HC, healthy control; SD, standard deviation.

As shown in Figure 2, one-way ANOVA revealed that there were significant differences in BFEIT performance (emotion identification accuracy) among the AT group, CATP group, and HC group (*F*_2,90_ = 27.729, *df* = 2, η_*p*_^2^ **=** 0.33, *p* = 0.000). Least square difference tests were performed as *post hoc* analyses and showed significant differences between the HC group, AT group, and CATP group (all *p* = 0.000). The emotion identification accuracy of the HC group was higher than that of the AT group or CATP group. However, no significant difference was observed between the AT group and the CATP group (*p* = 0.951).

### Comparisons of HAMD (17-Item Version) Scores Before and After Clinical Interventions

**Figure 3:**
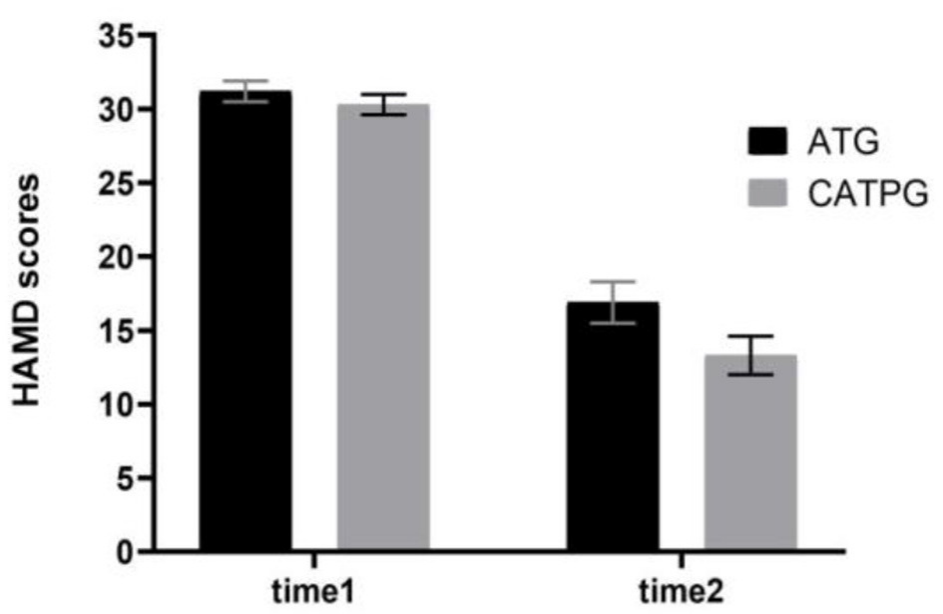
Comparisons of HAMD scores before and after clinical interventions between the AT group and CATP group. HAMD, Hamilton Depression Rating Scale (17-item version); ATG, antidepressant treatment group; CATPG, the combination of antidepressant treatment and PST group; time 1, baseline; time 2, after 12 weeks of intervention; SD, standard deviation.

As shown in Figure 3, using HAMD (17-item version) scores as dependent variables, a 2 × 2 repeated-measures ANOVA with group (AT group *vs*. CATP group) as a between-subjects factor and time point (time 1 *vs*. time 2) as a within-subjects factor revealed that the interaction effect for group × time point was not significant (*F*_1,61_ = 1.697, η_*p*_^2^ **=** 0.003, *p* = 0.198); however, the main effect for time point was significant (*F*_1,61_ = 206.419, η_*p*_^2^ **=** 0.35, *p* = 0.000), and the main effect for group was significant (*F*_1,61_ = 170.914, η_*p*_^2^ **=** 0.18, *p* = 0.038). The 12-week interventions decreased HAMD (17-item version) scores in the two patient groups.

There were significant differences in the remission rate between the CATP group (19/32) and the AT group (14/31); the remission rate in the CATP group was higher than that of the AT group (χ^2^ = 6.123, ϕ = 0.29, *p* = 0.028). There were significant differences in the treatment response rate between the CATP group (25/32) and AT group (18/31); the treatment response rate in the CATP group was higher than that of the AT group (χ^2^ = 4.370, ϕ = 0.26, *p* = 0.035).

## Comparisons of FVT Performance Among the AT Group, CATP Group, and HC Group

### Baseline Level

As shown in Table 2, one-way ANOVA revealed that there were significant differences in face response proportions and vignette response proportions among the AT group, CATP group, and HC group (*F*_2,90_ = 27.861, 18.234, all *df* = 2; η_*p*_^2^ **=** 0.32, 0.36, all *p* = 0.000). Least square difference tests were performed as *post hoc* analyses and showed significant differences between the HC group and AT group or between the HC group and the CATP group (all *p* = 0.000). The face response proportions of the HC group were lower than those of the AT group and CATP group, and the vignette response proportions of the HC group were higher than those of the AT group and CATP group. For the above-mentioned two variables, no differences between the AT group and CATP group were observed (*p* = 0.951, 0.913).

**Table 2:** Face–vignette task performances (%, SD) among the AT group, CATP group, and healthy control group.

	**AT group (*****n*** **=** **31)**			**CATP group (*****n*** **=** **32)**			**HC group (*****n*** **=** **30)**		
	**F**	**V**	**R**	**F**	**V**	**R**	**F**	**V**	**R**
Time 1	38 (7)	58 (9)	5 (3)	38 (7)	57 (9)	5 (3)	24 (1)	70 (5)	6 (3)
Time 2	32 (8)	63 (1)	5 (3)	25 (8)	71 (1)	4 (4)	–	–	–

*AT, antidepressant treatment; CATP, the combination of antidepressant treatment and PST; Time 1, baseline; Time 2, after 12 weeks of interventions; F, face response proportions; V, vignette response proportions; R, random response proportions*.

However, there were no significant differences in random response proportions among the AT group, CATP group, and HC group (*F*_2,90_ = 0.979, *df* = 2, η_*p*_^2^ **=** 0.006, *p* = 0.380).

### Before and After Interventions

As shown in Table 2, using face response proportions, vignette response proportions, and random response proportions as dependent variables, a 2 × 2 repeated-measures ANOVA with group (AT group *vs*. CATP group) as the between-subjects factor and time point (time 1 *vs*. time 2) as the within-subjects factor was performed.

### Face Response Proportions

The interaction effect for group × time point was significant (*F*_1,61_ =25.174, *df* =1, η_*p*_^2^ **=** 0.30, *p* = 0.000), the main effect for time point was significant (*F*_1,61_ = 138.086, *df* = 1, η_*p*_^2^ **=** 0.32, *p* = 0.000), and the main effect for group was significant (*F*_1,61_ = 4.853, *df* = 1, η_*p*_^2^ **=** 0.24, *p* = 0.031).

### Vignette Response Proportions

The interaction effect for group × time point was significant (*F*_1,61_ = 29.450, *df* = 1, η_*p*_^2^ **=** 0.31, *p* = 0.000), the main effect for time point was significant (*F*_1,61_ = 144.130, *df* = 1, η_*p*_^2^ **=** 0.32, *p* = 0.000), and the main effect for group was significant (*F*_1,61_ = 3.083, *df* = 1, η_*p*_^2^ **=** 0.18, *p* = 0.041).

### Random Response Proportions

The interaction effect for group × time point was not significant (*F*_1,61_ = 1.003, *df* = 1, η_*p*_^2^ **=** 0.001, *p* = 0.320), the main effect for time point was not significant (*F*_1,61_ = 1.519, *df* = 1, η_*p*_^2^ **=** 0.001, *p* = 0.223), and the main effect for group was not significant (*F*_1,61_ = 0.017, *df* = 1, η_*p*_^2^ **=** 0.000, *p* = 0.897).

## Discussion

This study is the first to survey the effect of problem-solving therapy on effortful cognition in MD using FVT; measurements of the basic facial emotion identification were also conducted. Our data showed that the emotion identification accuracy of HCs was higher than that of patients with MD; patients with MD exhibited poor FVT performance. Compared to antidepressant treatment, PST plus antidepressant treatment resulted in lower HAMD (17-item version) scores and better FVT performance.

This study also investigated the ability of patients with MD to employ contextual information when determining the intended or expressed or signified message of facial emotional expressions. In the FVT, target facial emotional expressions are preceded by stories describing situational messages which are discrepant in affective valence. What both patients with MD and HCs had judged reflects either the dominance of the emotional context or the facial emotional expression. Many studies on cognitive processing by patients with MD reported that depressive symptoms interfere with effortful processing, and the degree of interference is determined by the degree of effort required for the task, the severity of depression, and the valence of the stimulus material to be processed. However, depressive symptoms only interfere minimally with automatic processes (27).

Consistent with the findings of previous studies (21–23), our results showed that patients with MD could not utilize contextual information for specific face–vignette pairs. However, HCs more extensively made good judgments on emotion in line with contextual information, which indicates that patients with MD display poor effortful cognition performance. Cognition dysfunctions in MD include impairments of social cognition and neurocognition (28, 29). Social cognition refers to a process or a function for an individual's mental operations underlying social behavior, while neurocognition refers to those basic information processing functions such as attention and executive processes. Effortful cognitive processing was involved in either social cognition or neurocognition. We verified our hypothesis, *i.e*., patients with MD present effortful cognitive dysfunction.

In this study, we confirmed that PST plus antidepressant treatments leads to a greater reduction of depressive symptoms, a greater response rate, and a greater remission rate over a period of 12 weeks than antidepressant treatments only in patients with MD. We also indirectly verified our previous hypothesis, *i.e*., PST can improve effortful cognitive dysfunction, namely, PST improved the severity of MD by improving effortful cognition. Our data provide supporting evidence for the conclusion that the facial affect processing ability could be a valuable predictor of successful social context integration in FVT in MD.

## Conclusions

In conclusion, patients with MD present effortful cognitive dysfunction, and PST can improve effortful cognitive dysfunction. The measurement of effortful cognition might be one of the indexes for the therapeutic effect of PST in MD.

There are some limitations in the study. First, the findings must be considered preliminary due to the small sample size. Second, healthy controls were assessed with the FVT only once; therefore, the results of the FVT would be influenced by the practice effect in patients with MD. Future studies should augment the sample size and eliminate the practice effect to further confirm the relationship between effortful cognition and PST in MD. Finally, this study investigated the effect of PST plus antidepressant treatment on effortful cognition in MD. Therefore, no outcome of the pure PST effect on effortful cognition was obtained. The examination of the pure PST effect on effortful cognition in MD is necessary in a future study.

## Data Availability Statement

The datasets generated for this study are available on request to the corresponding author.

## Ethics Statement

The studies involving human participants were reviewed and approved by Affiliated Wuxi Mental Health Center of Nanjing Medical University. The patients/participants provided their written informed consent to participate in this study.

## Author Contributions

CJ, HZ, and ZZ designed the study and wrote the paper. CJ, HZ, LC, and ZZ acquired and analyzed the data. All authors reviewed the content and approved the final version for publication.

## Conflict of Interest

The authors declare that the research was conducted in the absence of any commercial or financial relationships that could be construed as a potential conflict of interest.
